# Observational Study of the Association between Hyponatremia and Rhabdomyolysis in Patients Presenting to Hospital

**DOI:** 10.3390/jcm11113215

**Published:** 2022-06-05

**Authors:** Andy K. H. Lim, Ajinkya Bhonsle, Karen Zhang, Joy Hong, Kuo L. C. Huang, Joseph Nim

**Affiliations:** 1 Department of General Medicine, Monash Health, Clayton, VIC 3168, Australia; ajinkya.bhonsle@monashhealth.org (A.B.); karen.zhang@monashhealth.org (K.Z.); joy.hong@monashhealth.org (J.H.); kuolincolin.huang@monashhealth.org (K.L.C.H.); joseph.nim@monashhealth.org (J.N.); 2 Department of Nephrology, Monash Health, Clayton, VIC 3168, Australia; 3 Department of Medicine, Monash University School of Clinical Sciences, Clayton, VIC 3168, Australia

**Keywords:** sodium, hyponatremia, hypoosmolality, rhabdomyolysis, muscle injury, creatine kinase

## Abstract

Hyponatremia may be a risk factor for rhabdomyolysis, but the association is not well defined and may be confounded by other variables. The aims of this study were to determine the prevalence and strength of the association between hyponatremia and rhabdomyolysis and to profile patients with hyponatremia. In a cross-sectional study of 870 adults admitted to hospital with rhabdomyolysis and a median peak creatine kinase of 4064 U/L (interquartile range, 1921–12,002 U/L), glucose-corrected serum sodium levels at presentation showed a U-shape relationship to log peak creatine kinase. The prevalence of mild (130–134 mmol/L), moderate (125–129 mmol/L), and severe (<125 mmol/L) hyponatremia was 9.4%, 2.5%, and 2.1%, respectively. We excluded patients with hypernatremia and used multivariable linear regression for analysis (*n* = 809). Using normal Na^+^ (135–145 mmol/L) as the reference category, we estimated that a drop in Na^+^ moving from one Na^+^ category to the next was associated with a 25% higher creatine kinase after adjusting for age, alcohol, illicit drugs, diabetes, and psychotic disorders. Multifactorial causes of rhabdomyolysis were more common than single causes. The prevalence of psychotic and alcohol use disorders was higher in the study population compared to the general population, corresponding with greater exposure to psychotropic medications and illicit drugs associated with hyponatremia and rhabdomyolysis. In conclusion, we found an association between hyponatremia and the severity of rhabdomyolysis, even after allowing for confounders.

## 1. Introduction

In humans, serum sodium concentration (Na^+^) is maintained between 135 mmol/L and 145 mmol/L. Hyponatremia occurs due to an imbalance between water and sodium, such as the depletion of body sodium more than body water or the dilution of sodium due to the retention of body water more than body sodium. The extracellular fluid volume may be reduced (hypovolemia), normal (euvolemia), or elevated (hypervolemia). Hyponatremia is the most common fluid–electrolyte disorder in clinical practice. Mild hyponatremia is often asymptomatic, but moderate (Na^+^ < 130 mmol/L) or severe hyponatremia (Na^+^ < 125 mmol/L) is more likely to be associated with complications, particularly if it develops rapidly. Elderly patients are prone to hyponatremia and its complications. The typical symptoms are mainly neurological, such as lethargy, headache, confusion, seizures, and muscle weakness. Patients with severe hyponatremia may develop encephalopathy due to brain edema. However, one uncommon syndrome associated with hyponatremia is rhabdomyolysis.

Rhabdomyolysis is a clinical syndrome of injury to muscle cells, which results in the release of toxic substances into the circulation, leading to secondary injury in other organs. These harmful substances include high levels of potassium, phosphate, and myoglobin, which may cause cardiac instability, acute kidney injury (AKI), or disseminated intravascular coagulation. The hallmark of rhabdomyolysis is a significantly elevated serum creatine kinase (CK).

There have been many case reports of rhabdomyolysis associated with hyponatremia [1,2,3,4]. The exact nature of the association between low Na^+^ and rhabdomyolysis is unclear and may be confounded by other processes. Whether or not severe hyponatremia is a direct causal factor for rhabdomyolysis is not clear. One example of a complicated relationship is the effects of illicit drugs such as ecstasy. Ecstasy is associated with both hyponatremia and rhabdomyolysis. However, rhabdomyolysis may be due to drug-induced seizures, malignant hyperthermia, or pressure injuries from prolonged immobilization, and not directly due to a low Na^+^ [5,6]. Other studies have suggested that the rapid correction of hyponatremia is associated with rhabdomyolysis rather than hyponatremia per se [7]. Even though there is a proof-of-concept clinical study that showed that serum Na^+^ could drop before CK rises in marathon runners [8], there are no convincing animal studies that duplicate these observations in humans to demonstrate causality [9]. Another prospective human study was not able to show a clear association between ultra-running, hyponatremia, and rhabdomyolysis [10].

The aims of this study were: (1) to determine the prevalence of hyponatremia in patients presenting to the hospital with rhabdomyolysis; (2) to determine the profile of patients with significant hyponatremia; and (3) to examine the strength of the association between hyponatremia and rhabdomyolysis, while accounting for potential confounders.

## 2. Methods

### 2.1. Study Design and Setting

We conducted a cross-sectional study of patients admitted to three acute care hospitals at Monash Health, a tertiary referral healthcare network in Australia (Monash Medical Centre, Dandenong Hospital, Casey Hospital), from October 2015 to August 2021. The Monash healthcare network provides healthcare services to around a quarter of the population of the city of Melbourne, in the southeast of the state of Victoria.

### 2.2. Study Population

We used the International Statistical Classification of Diseases and Related Health Problems, Tenth Revision (ICD-10) codes to identify adult patients (≥17 years) admitted to hospital with muscle injury and systematically examined the health records to confirm a clinical and biochemical diagnosis of rhabdomyolysis. Ambulatory care patients (‘outpatients’) were not eligible. For a diagnosis of rhabdomyolysis, the serum CK must be elevated at least 5 times above the reference range (>1000 U/L), and the patient has a clinical syndrome compatible with rhabdomyolysis. Patients were excluded if the (1) admission data were not available (e.g., transfer of care or retrieval from another health service); (2) CK rise was due to isolated myocardial injury, such as myocarditis and massive myocardial infarction. The identification of myocardial injury is based on standard clinical grounds: history of angina, elevated cardiac troponin biomarker and ECG findings, supplemented by echocardiography; (3) patient presented following cardiac arrest; (4) entries were duplicated due to the transfer of patients from the initial acute hospital to another site for specialized care or rehabilitation.

### 2.3. Hyponatremia and Other Biochemistry

We defined hyponatremia using the clinical practice guidelines of the Hyponatremia Guideline Development Group, endorsed by the European Society of Endocrinology, European Society of Intensive Care Medicine, and European Renal Association—European Dialysis and Transplant Association [11]. Na^+^ levels were classified as normal (Na^+^ 135–145 mmol/L), mild hyponatremia (Na^+^ 130–134 mmol/L), moderate hyponatremia (Na^+^ 125–129 mmol/L), and severe hyponatremia (Na^+^ < 125 mmol/L). We did not specifically define acute versus chronic hyponatremia as it was not possible to determine if the onset was <48 h prior to presentation in most cases. To correct Na^+^ for serum glucose levels, we added 2.4 mmol/L to the measured Na^+^ concentration for every 5.6 mmol/L (100 mg/dL) incremental rise in serum glucose concentration above 5.6 mmol/L [11]. To avoid missing data for glucose, we accepted both blood glucose and fingerprick capillary measurements. For the remainder of this article, Na^+^ refers to the glucose-corrected values unless explicitly stated as raw (uncorrected) values. We also collected data on serum calcium, phosphate, magnesium, and potassium, as severe deficiency of these electrolytes has been linked to rhabdomyolysis.

Electrolyte data were obtained from the first biochemistry test at presentation to hospital. Where calcium, phosphate, or magnesium was not available at presentation, we accepted measurements performed within 24 h of the initial biochemistry as long as no specific treatment for electrolyte abnormalities had been administered in the interim. In patients with Na^+^ < 130 mmol/L, we also collected data on serum osmolality. All patients had serial CK tests, and we used the peak CK for analysis. In 67% of patients, the peak CK corresponded with the first biochemistry test at presentation. In 22% of patients, the peak CK was recorded <24 h after admission. In the remaining 11% of patients, peak CK was recorded between 24 h and 72 h of admission.

### 2.4. Other Variable Definitions

We collected comorbidity data as binary variables, with the exception of mental health disorders. For this study, chronic kidney disease (CKD) was defined by an estimated glomerular filtration rate (eGFR) < 60 mL/min/1.73 m^2^ on at least two occasions 90 days apart, as reported by Australian laboratories using the CKD-Epidemiology Collaboration (CKD-EPI) equation. Baseline kidney function and eGFR were determined by the strategy recommended by Siew et al. [12]. For 95% of patients, we used the mean outpatient serum creatinine values measured 7–365 days prior to admission to calculate baseline eGFR. In 5% of patients where outpatient creatinine values were unavailable, we used the final creatinine from a prior inpatient admission. AKI was defined using the serum creatinine criteria of the Kidney Disease Improving Global Outcomes (KDIGO) guidelines for AKI [13]. In brief, Stage 1 AKI is an increase in serum creatinine ≥ 0.3 mg/dl (27 µmol/L) above baseline within 48 h, or increase ≥ 1.5 times baseline; Stage 2 is a serum creatinine increase 2.0–2.9 times baseline; and Stage 3 is a serum creatinine increase 3.0 times baseline or increase to ≥4.0 mg/dl (≥354 µmol/L), or having to initiate renal replacement therapy. The presence of CKD was verified by a nephrologist (A.K.H.L.). We used the guidelines of the Sepsis-3 report to determine the presence of sepsis syndrome [14].

### 2.5. Etiology of Rhabdomyolysis

Investigators performed a thorough analysis of the medical record to determine potential causes of rhabdomyolysis. We reviewed the ambulance, emergency department, and medical admission notes. Allied health, psychiatry, and toxicology assessments were examined if relevant. Regular medications were verified with dispensing pharmacies. Where uncertainty exists, a group consensus was achieved by discussion. Investigators were calibrated by instructional video, and a periodic review of data entry was undertaken by the principal investigator (A.K.H.L.). The etiological categories were not mutually exclusive, and the premise of this study was that multiple contributors were possible. However, we attempted to assign a single category of causation if there was only one dominant mechanism that was deemed ‘definite’, and only assigned more than one category if a second or third mechanism was deemed at least ‘probable’. For the purpose of this study, potential causes deemed ‘possible’ and ‘unlikely’ were not considered. A description of the categories is shown in Supplementary Table S1.

### 2.6. Statistical Analysis

We report the mean and standard deviation (SD) for data with normal distribution, and used the median and interquartile range (IQR) to describe data with skewed distribution. To compare means between two or more groups, we used a t test or one-way analysis of variance. To compare equality of distributions between Na^+^ groups, we used the Mann–Whitney test or Kruskal–Wallis with Dunn’s multiple-comparison test. As an adjunct to the Kruskal–Wallis test, we also performed a nonparametric test for linear trends across ordinal categories.

To initially explore and model the relationship between Na^+^ and CK as continuous variables, we used fractional polynomials to determine linearity and search for the best combination of powers. Then, we parameterized Na^+^ according to the categories of hyponatremia severity. We performed a log transformation of CK due to the highly skewed distribution and conducted linear regression analysis of log CK on hyponatremia categories as the main exposure (explanatory) variable. We examined the baseline differences in patients by hyponatremia categories and determined variables associated with both CK and Na^+^. Univariable regression was performed on variables demonstrating significant baseline differences and potential confounders. Next, we used a purposeful selection method and stepwise approach with forward addition for multivariable regression. We retained variables that were statistically significant or changed the *b* coefficient of the exposure variable by 10%. A *p* < 0.05 was considered statistically significant. All analyses were conducted using Stata version 16 (StataCorp, College Station, TX, USA).

## 3. Results

### 3.1. Study Population and Serum Sodium

**Figure 1:**
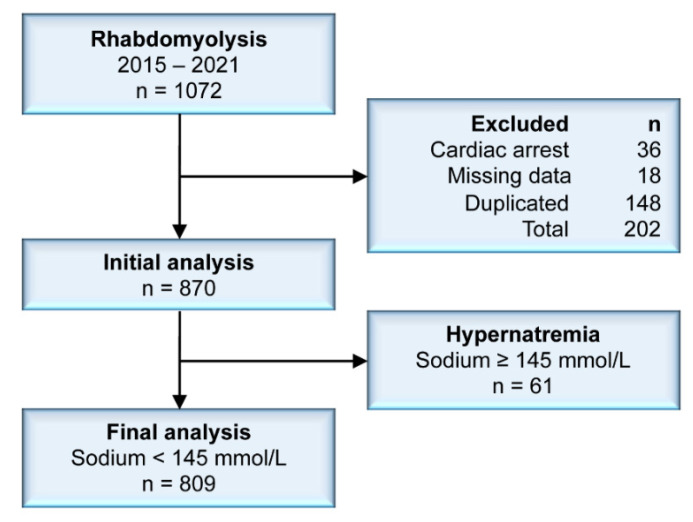
Study flow diagram. Note: sodium corrected for glucose levels.

**Figure 2:**
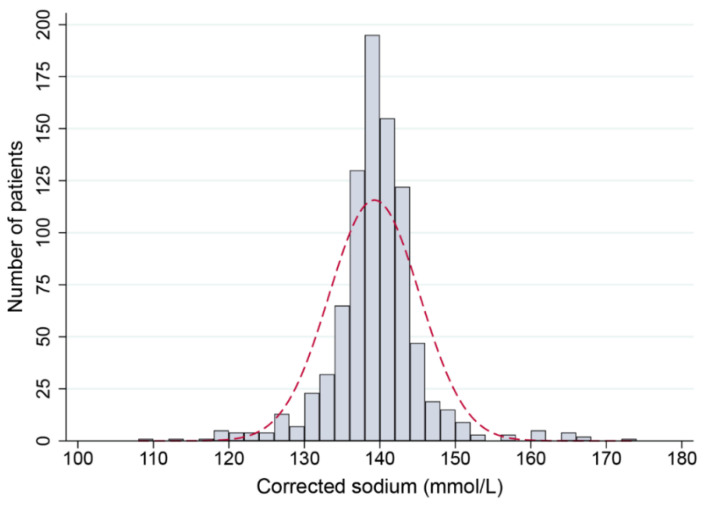
Histogram of admission serum sodium concentrations (corrected for glucose levels) with superimposed normal distribution curve (dashed red line). Each bar represents a 2 mmol/L interval. The peaked distribution curve suggested that severe hypo- or hypernatremia was uncommon.

We included 870 patients in the initial analysis. A flow diagram of patient selection and exclusions is shown in Figure 1. The distribution of age was skewed to the left (skewness, −0.81). The mean age was 67 years, but the median was 75 years. The male-to-female ratio was 1:1.3. At presentation with rhabdomyolysis, Na^+^ showed a distribution that was non-skewed but leptokurtotic, indicating that severe dysnatremias were uncommon (Figure 2). The mean Na^+^ was 139.3 mmol/L (SD, 6.0 mmol/L). The prevalence of mild, moderate, and severe hyponatremia was 9.4%, 2.5%, and 2.1%, respectively. For hypernatremia, a Na^+^ of 146–159 mmol/L (mild) was observed in 1.7% of patients, Na^+^ 160–169 mmol/L (moderate) in 1.4% of patients, and 0.1% had a Na^+^ ≥ 170 mmol/L (severe).

### 3.2. Baseline Patient Characteristics

**Figure 3:**
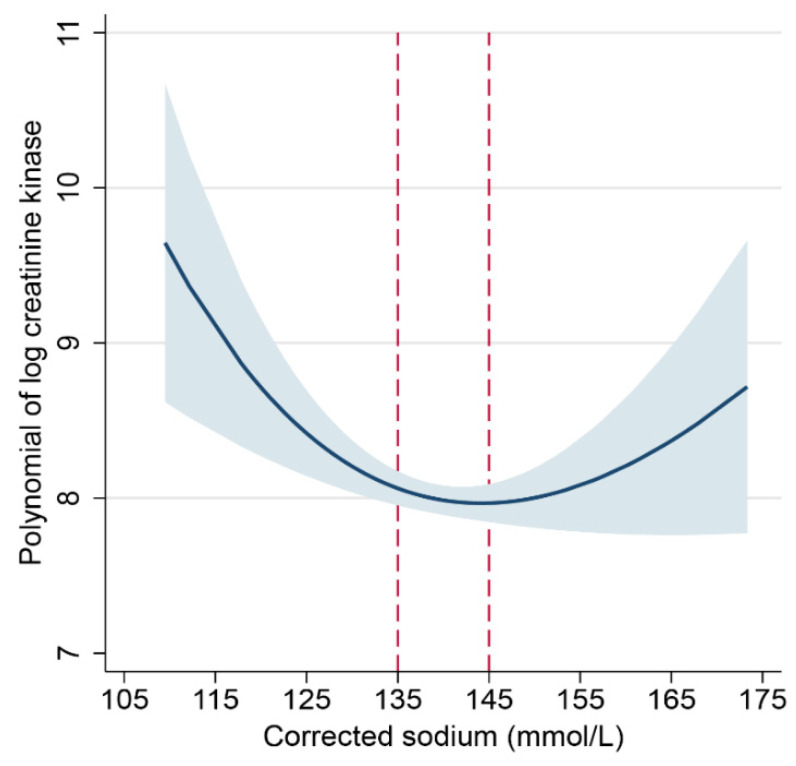
Regression of creatine kinase on sodium using fractional polynomials (blue line) with 95% confidence intervals (blue-gray band). The best-fitting relationship was U-shaped, suggesting that hypo- or hypernatremia was associated with higher creatine kinase, but a steeper left side suggested a larger effect of severe hyponatremia. Values in between the dashed red lines represent normal sodium levels (reference range).

To initially clarify the relationship between hyponatremia and the severity of rhabdomyolysis, we used the natural log-transformed peak CK as a surrogate for the severity of rhabdomyolysis (Supplementary Figure S1). Then, we used fractional polynomials to model the relationship between Na^+^ and log peak CK. This regression plot showed a non-linear, U-shaped relationship, with both low and high Na^+^ associated with a higher CK compared to normal Na^+^ (Figure 3). The upward trend of the curve with Na^+^ > 145 mmol/L suggested that patients with Na^+^ levels above this level may not be comparable to patients with Na^+^ of 135–145 mmol/L.

For the purpose of this study, hypernatremic patients were discarded in further analysis. Thus, we parameterized Na^+^ as an ordinal variable in further analysis of the association between Na^+^ and CK, using patients with normal Na^+^ (135–145 mmol/L) as the reference group.

Table 1 summarizes the baseline characteristics, grouped by hyponatremia status at presentation. Given the older age of the study population, 23% had diabetes, 18% had ischemic heart disease, 20% patients had CKD, and 11% had chronic lung disease. Only 8% of patients had established dementia and 4% lived in a residential aged care facility. The prevalence of mood and anxiety disorders in this study is similar to the 10–13% in the general Australian population, but the prevalence of psychotic illness observed in this study population was much higher than the 0.4% estimated for adults aged 18–64 years in our community [15,16]. The prevalence of illicit drug use in our study population was similar to the 15.9% to 16.8% reported for people aged 18 and over in 2016–2019 for the general population of Australia [17].

Comparing the age distribution, patients with Na^+^ < 130 mmol/L were younger than patients with Na^+^ 130–145 mmol/L (*p* = 0.029). Following this age variation, patients with Na^+^ < 130 mmol/L had a lower burden of ischemic heart disease (5.0% vs. 19.1%, *p* = 0.021) and CKD (0% vs. 21.3%, *p* < 0.001) as well. Comparing the Na^+^ groups, we did not find a significant difference in strokes (*p* = 0.13), peripheral vascular disease (*p* = 0.93), heart failure (*p* = 0.67), or active cancer (*p* = 0.81), but the prevalences of these conditions were low overall. However, the proportion of patients with diabetes differed between the Na^+^ groups (*p* = 0.037). Compared to the reference group, there was a low prevalence amongst patients with mild hyponatremia (13.4% vs. 24.5%, *p* = 0.028) and severe hyponatremia (5.6% vs. 24.5%, *p* = 0.08).

Excess alcohol use was strongly associated with Na^+^ levels (*p* < 0.001), which took the form of a linear trend across the ordinal Na^+^ categories (z = 4.02, *p* < 0.001), with the proportion of patients drinking excessively increasing from 9.5% to 38.9%, moving from normal Na^+^ to severe hyponatremia. Illicit drug use was associated with Na^+^ levels, with a higher proportion of patients using illicit drugs in those with Na^+^ < 130 mmol/L compared to those with Na^+^ 130–145 mmol/L (22.5% vs. 11.4%, *p* = 0.036). Finally, psychotic disorders were significantly more prevalent in those with Na^+^ < 130 mmol/L than those with Na^+^ 130–145 mmol/L (22.5% vs. 6.1%, *p* < 0.001).

### 3.3. Rhabdomyolysis, Biochemistry, and Complications

Biochemistry and clinical data are summarized in Table 2. The distribution of CK was skewed to the right, with a median of 4064 U/L (IQR, 1921 to 12 002 U/L). Analysis of the distribution of CK demonstrated that CK was higher in patients with Na^+^ < 130 mmol/L compared to patients with Na^+^ 130–145 mmol/L (*p* = 0.016), and the mean difference in log CK between these two groups was 0.68 log (95% CI: 0.11 to 1.25, *p* = 0.02) at a Na^+^ threshold of 130 mmol/L.

**Figure 4:**
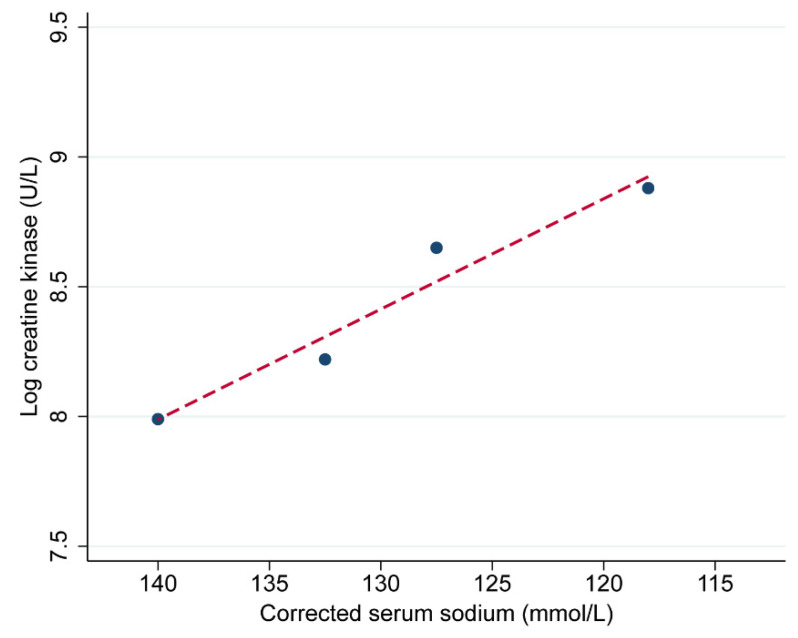
Plot of the mean log creatine kinase versus the mid-point of sodium categories, and fitted with least-squares linear regression line, demonstrating a linear trend for increasing creatine kinase moving normal sodium concentration to severe hyponatremia. Note: scale is reversed on the x-axis, with sodium decreasing from left to right.

There was also a positive linear trend in log CK, with each categorical change in Na^+^ moving across from normal Na^+^ levels to severe hyponatremia (Figure 4). In later analysis, we further examined the effect of this categorical change on CK as well as the effect at a Na^+^ threshold of 130 mmol/L. There was no significant difference in potassium, phosphate, magnesium, or glucose between patients across the Na^+^ categories. However, there were differences in serum calcium between the Na^+^ groups at presentation (*p* < 0.001). When compared to the reference group, mean serum calcium was 0.06 mmol/L lower in patients with mild hyponatremia (*p* = 0.022), and 0.16 mmol/L lower in patients with moderate (*p* < 0.001) and severe hyponatremia (*p* = 0.001).

There were minor differences in the distribution of peak serum creatinine between the Na^+^ groups (*p* = 0.03) due to the differences in CKD between the Na^+^ categories. It was likely that the observed differences in peak creatinine reflect a better baseline kidney function on average in patients in the severe hyponatremia category. More importantly, there were no significant differences in the proportion of patients with AKI (*p* = 0.37), needing renal replacement therapy (*p* = 0.71), or experiencing sepsis syndrome (*p* = 0.27). There was no significant difference in hospital length of stay based on Na^+^ categories (*p* = 0.58), but there was a higher proportion of patients with severe hyponatremia admitted to intensive care compared to all other patients (61.1% vs. 21.6%, *p* < 0.001).

### 3.4. Etiology of Hyponatremia

For patients with Na^+^ < 130 mmol/L, we reported the contributors to hyponatremia determined by treating clinicians, corroborated urinary and serum Na^+^ and osmolality, prior history of hyponatremia, medications prior to presentation, and the trajectory of hyponatremia (Table 3). Multifactorial causes were present in 55% of patients (22 of 40). Hypovolemia was present in half, and almost one-third were drinking alcohol in excess or binge drinking, including one patient who was drinking 18 beers daily (beer potomania). Antipsychotics (e.g., flupentixol, olanzapine, zuclopenthixol, quetiapine), antidepressants (e.g., citalopram, sertraline, venlafaxine, mirtazapine, amitriptyline), illicit drugs (methamphetamines (ice, crystal meth, ecstasy), tetrahydrocannabinol, methadone, heroin, cocaine), and diuretics (thiazides) were frequently implicated. Less common contributors were antiepileptics (e.g., valproate, carbamazepine, levetiracetam), gastrointestinal losses, cerebral salt wasting, and ineffective arterial blood volume states, such as severe heart failure or advanced liver cirrhosis. There were more patients with primary polydipsia in the group with Na^+^ < 125 mmol/L compared to the Na^+^ 125–129 mmol/L group (33% vs. 5%, *p* = 0.033), contrasting with the higher proportion of patients with a syndrome of inappropriate antidiuretic hormone (SIADH) in the Na^+^ 125–129 mmol/L group (6% vs. 32%, *p* = 0.01). Our profiling implies that moderate to severe hyponatremia was often associated with illicit drugs and alcohol misuse, regular use or overdose of psychotropic (antipsychotic or antidepressant) medications, and that polydipsia may be an additional risk factor for severity.

### 3.5. Mechanism of Rhabdomyolysis

A summary of the causes of rhabdomyolysis is shown in Table 4. The commonest factors associated with rhabdomyolysis were pressure injury due to prolonged immobilization and drugs/toxins, followed by infection, trauma, and exertional injury. Patients with severe hyponatremia demonstrated a different profile of associated causes. Compared to the other Na^+^ categories, patients with Na^+^ < 125 mmol/L had no cases linked to trauma, ischemia, or exertion and fewer cases linked to pressure injuries from prolonged immobilization. Instead, seizures were over-represented in the severe hyponatremia group. Patients with Na^+^ < 125 mmol/L were more likely to present with witnessed seizures compared to the other groups (33.3% vs. 3.1%, *p* < 0.001), and this association showed a small to medium effect size (Cramer’s V = 0.25).

The most commonly encountered drugs and toxins in descending order of frequency were antipsychotic medications (20%); followed closely by heroin, methadone, and other opioids (18%); meth/amphetamines (17%); ethanol intoxication (15%); selective serotonin- or serotonin–norepinephrine reuptake inhibitors (7%); and tetrahydrocannabinol or synthetic cannabinoids (6%). Less commonly encountered, at 2% to 3% rate of prevalence, were cocaine, gamma hydroxybutyrate (GHB), ecstasy (MDMA), lysergic acid diethylamide (LSD), wasp or bee stings, and tiger snake envenomation.

The overall prevalence of statin use was 32.4%, but we estimated that only 0.9% of cases of rhabdomyolysis could convincingly be attributed to statins, based on a lack of other contributors and improvement in CK on cessation. Most cases of thermal extremes were due to hypothermia (n = 18), with a mean body temperature of 29.7 °C (SD, 2.3 °C), but five cases were due to heatstroke. Inflammatory myopathies, inherited conditions, and electrolyte derangements other than hyponatremia were rare (<1%). We identified four cases of severe hypokalemia (1.3,1.5, 2.2, and 2.4 mmol/L), one of which was associated with Na^+^ of 118 mmol/L, one case of severe hypophosphatemia (<0.20 mmol/L), and one of severe hypomagnesemia (0.30 mmol/L).

### 3.6. Regression Analysis

**Figure 5:**
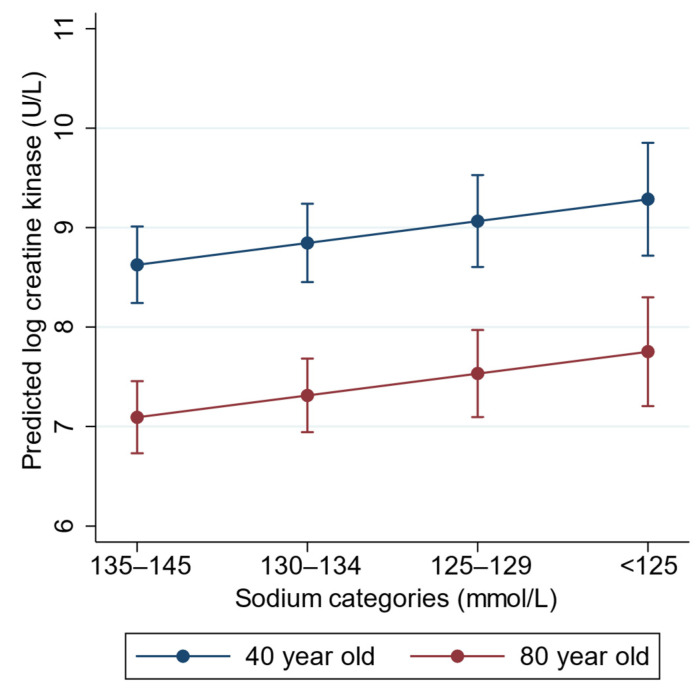
Predicted creatine kinase from regression model with adjusted means and 95% confidence intervals, showing higher creatine kinase with increasing hyponatremia severity. Creatine kinase was lower in elderly patients at the same level as the other covariates.

Next, we investigated the univariable regression of log peak CK on the exposure factor (Na^+^) and other variables with important baseline differences between Na^+^ groups or potential confounders (Table 5). Then, we proceeded to multivariable modeling (Table 6). Age demonstrated the most significant confounding effect, with a 23% change in the *b* coefficient of the exposure variable after inclusion in the model. After adjusting for age, the effects of CKD and pressure injury were no longer significant. Age and pressure injury were highly correlated (r = 0.56, *p* < 0.001), while age and CKD were moderately correlated (r = 0.38, *p* < 0.001). Thus, CKD and pressure variables were omitted from the multivariable model. Most seizures appeared to be on the causal pathway between hyponatremia and rhabdomyolysis and were therefore not considered as a confounder and omitted from the model. Alcohol and illicit drugs were both significantly correlated with the exposure variable and CK levels and thus were potential confounders. The addition of alcohol and illicit drugs changed the *b* coefficient by 15%. Similarly, diabetes and psychotic disorders were significantly correlated with both Na^+^ levels and CK levels, and the inclusion of diabetes and psychotic disorders changed the *b* coefficient by 11%. In the final model, on average, every category increase in hyponatremia severity to the next was associated with a 0.22 log higher peak CK level (95% CI: 0.03 to 0.41, *p* = 0.024) after adjusting for age, alcohol and illicit drugs, psychotic disorders, and diabetes. There were no significant statistical interactions between the covariates. Plots of the marginal predictions from the final model are shown in Figure 5, and regression diagnostic plots are presented in Supplementary Figure S2.

### 3.7. Other Analysis

We performed a sensitivity analysis, excluding patients whose peak CK was not derived from the first biochemistry test at presentation (CK continued to rise after presentation). In this analysis using the same covariates (n = 538), the *b* coefficient for the exposure variable increased from the previous estimate, to 0.48 (95% CI: 0.21 to 0.71, *p* < 0.001). To determine possible selection bias, we examined the characteristics of the 61 patients (7%) excluded with hypernatremia. Their median age was 77 years (IQR, 54 to 84 years), and 48% were male. The major comorbidities were similar to the included patients, with the exception of a higher prevalence of diabetes (38%) and ischemic heart disease (30%). Correspondingly, average blood glucose was higher at a median of 8.8 mmol/L (IQR, 6.6 to 18.2 mmol/L). There was a greater prevalence of sepsis (16%), AKI (67%), renal replacement therapy (7%), and intensive care unit admission (39%). Illness severity was higher in those with Na^+^ > 150 mmol/L (n = 19), with a 53% prevalence of diabetes, 42% presenting with diabetic ketoacidosis or hyperosmolar hyperglycemia syndrome, and median glucose of 19.8 mmol/L (IQR, 8.8 to 52.0 mmol/L). Rates of complications were much higher, including sepsis (26%), AKI (89%), renal replacement therapy (16%), and intensive care unit admission (63%).

## 4. Discussion

In this study of patients presenting to the hospital with a diagnosis of rhabdomyolysis, the prevalence of mild, moderate, and severe hyponatremia was 9.4%, 2.5%, and 2.1%, respectively. We used peak CK as a surrogate marker for the severity of rhabdomyolysis and found a U-shaped association between Na^+^ and log-transformed peak CK. Hypernatremia was less common, and we excluded patients with Na^+^ > 145 mmol/L from the final analysis to simplify modeling. Using Na^+^ 135–145 mmol/L as the reference category, we found an association between lower Na^+^ and higher CK levels. Profiling of patients with hyponatremia indicated that patients with psychotic disorders, the use of psychotropic (antipsychotic or antidepressant) medications, and excessive alcohol intake were over-represented. This finding is consistent with a systematic review of hyponatremia and excessive water intake, which noted that 52% of patients had schizophrenia-spectrum disorders and 42% were on psychotropic medications associated with hyponatremia [18].

From our regression model, we estimated that a drop in Na^+^ from one category to the next was associated with an average 0.22 log increase in peak CK after adjusting for age, alcohol, illicit drugs, diabetes, and psychotic disorders. This equates to a 25% increase in CK (in standard units, U/L) for every one-unit increase in hyponatremia severity, with covariates held constant. Age was a strong confounder of this association. In an 80-year-old diabetic patient with excess alcohol intake but no psychiatric disorder or illicit drug use, and a Na^+^ of 137 mmol/L, the estimated peak CK was log 7.09 U/L, but if he presented with a Na^+^ of 132 mmol/L, the estimated peak CK was log 7.31 U/L. We modeled a 40-year-old with identical *b* coefficients for the other covariates. At a Na^+^ of 137 mmol/L, the estimated CK was log 8.85, but at a Na^+^ of 132 mmol/L, this was log 9.07. As the outcome variable was log-transformed, while the relative differences in exponentiated log CK (to the original units) were the same for both these patients when moving from normal Na^+^ to mild hyponatremia, the absolute differences in the original units were markedly different. The effect of hyponatremia may be more evident in younger patients due to greater muscle mass.

There are laboratory data from animal experiments demonstrating that acute hyponatremia could directly cause skeletal muscle edema, and there is a lack of acute regulatory volume decrease in muscle in response to hyponatremia [19]. The association between hyponatremia and rhabdomyolysis in humans is mostly evidenced by case reports. However, there was a proof-of-concept study conducted on 15 ultramarathon runners who underwent serial biochemistry tests at checkpoints during a long-distance run. Water imbalance developed during the run, and patients with the lowest category of Na^+^ (<129 mmol/L) had the highest CK at subsequent checkpoints. The authors concluded that transient exercise-induced hyponatremia preceded and augmented the CK levels [8]. Perhaps hyponatremia increased the susceptibility of muscle cells to injury from repetitive contractions or impact trauma. We cannot be certain that hyponatremia per se, in the absence of other insults, caused rhabdomyolysis. In a laboratory study of mouse skeletal muscle, the effect of low Na^+^ was more apparent in muscle fatigued by high-frequency stimulation compared to non-fatigued muscle, with a loss of sarcolemma excitability, a lower capacity to generate action potentials, and reduced peak force [20]. Furthermore, nerve conduction and motor function impairment in patients with severe hyponatremia can be reversed with the correction of Na^+^ [21].

Our study supports the concept that hyponatremia may be a risk factor rather than an all-or-nothing trigger for rhabdomyolysis. We could independently correlate hyponatremia severity with CK levels, but many factors were associated with both hyponatremia and CK levels and thus confounded this association. The *b* coefficient for the exposure variable was reduced by 21% after allowing for these variables. For example, patients with psychotic disorders may have primary polydipsia and receive treatment with antipsychotics, which can lead to low Na^+^ and elevated CK [22]. Patients who drink excess alcohol are prone to hyponatremia [23] and may develop rhabdomyolysis through multiple mechanisms, such as ethanol toxicity, pressure injury through immobilization, seizures, or severe hypothermia. Furthermore, alcohol use disorder is associated with multiple electrolyte disturbances and deficiencies in trace elements [23,24]. Illicit drugs such as methamphetamine and 3,4-methylenedioxymethamphetamine have been shown to cause hyponatremia and rhabdomyolysis through multiple mechanisms such as pressure injury through immobilization, seizures, excessive exertion, and direct muscle toxicity [25,26]. In our study, it was uncommon for patients with moderate to severe hyponatremia to have a single mechanism of muscle injury.

There are studies suggesting that the rapid correction of hyponatremia may be a trigger for rhabdomyolysis. In a study of 22 patients with primary polydipsia, rhabdomyolysis was associated with the speed of Na^+^ correction and the level achieved [27]. Another observational study of 56 patients with water intoxication reported that patients with a median correction rate of 1.1 mmol/L/h in the first 24 h were more likely to develop rhabdomyolysis than patients with a median correction rate of 0.7 mmol/L/h [7]. However, 67% of our patients achieved peak CK at presentation, prior to any corrective efforts, so this potential mechanism was not relevant to them. Furthermore, our sensitivity analysis showed that the strength of the association was even stronger after excluding patients with a rise in CK after presentation. By excluding patients with a delayed peak in CK, we removed the patients who may have had significant fluid treatment after their initial presentation. Thus, although we did not address the issue of treatment-related rhabdomyolysis in this study, we demonstrated a clear association between untreated hyponatremia and higher levels of CK.

To our knowledge, this is the first study to examine the association between hyponatremia and rhabdomyolysis from all causes and provide insight into important mechanisms. The main limitation of our study was the cross-sectional design, so a causal inference was not possible. We were limited to observational studies, as inducing hyponatremia is unethical. Prospective cohort studies in which hyponatremia is not corrected are also unethical. We welcome further laboratory studies to demonstrate a direct causative effect. A larger number of patients with moderate to severe hyponatremia could have improved the precision of our estimates and allowed the analysis of more covariates to avoid residual confounding. Patients with decompensated diabetes were more likely to present with hypernatremia, unmasked by sodium correction, which may also be a mechanism for rhabdomyolysis warranting further study, but the number of such patients was too small to provide reliable estimates, and we excluded them to focus on hyponatremia patients. We were not able to account for the acuity of the hyponatremia in a cross-sectional study, and this may have implications on risk as cellular compensation in chronic hyponatremia may be as relevant as the severity. Finally, we did not include seizures or serum calcium as independent variables in the regression model. We believe that seizures lie on the causal pathway, and the small difference in serum calcium in patients with Na^+^ < 130 mmol/L compared to Na^+^ ≥ 130 mmol/L reflected the greater severity of rhabdomyolysis and calcium deposition [28], and was not confounding.

## 5. Conclusions

Hyponatremia was associated with the severity of rhabdomyolysis in patients presenting to the hospital, even after adjusting for confounding, particularly by age. This association is clinically relevant to patients with psychiatric and alcohol use disorders, as they represent patients at higher risk of moderate to severe hyponatremia who have multiple potential mechanisms for developing rhabdomyolysis. For clinical practice, it is prudent to monitor hyponatremia in these patients and apply corrective measures to avoid moderate or severe hyponatremia while awaiting studies to clarify the causal nature of this association.

## Figures and Tables

**Table 1:** Baseline patient characteristics at hospital presentation grouped by serum sodium concentration.

		Corrected Serum Sodium (mmol/L)			
Characteristic	Total	135–145	130–134	125–129	<125
	*n* = 809	*n* = 687	*n* = 82	*n* = 22	*n* = 18
Age, median (IQR), years	75 (54–84)	75 (54–84)	75 (55–85)	67 (35–76)	59 (51–81)
Male sex, *n* (%)	347 (42.9)	290 (42.2)	38 (46.3)	10 (45.5)	9 (50.0)
Residential aged care, *n* (%)	29 (3.6)	22 (3.2)	5 (6.1)	2 (9.1)	0 (0)
Dementia, (%)	64 (7.9)	57 (8.3)	4 (4.9)	2 (9.1)	1 (5.6)
Diabetes mellitus, *n* (%)	185 (22.9)	168 (24.5)	11 (13.4)	5 (22.7)	1 (5.6)
Ischemic heart disease, *n* (%)	149 (18.4)	132 (19.2)	15 (18.3)	1 (4.5)	1 (5.6)
Congestive heart failure, *n* (%)	61 (7.5)	55 (8.0)	5 (6.1)	0 (0)	1 (5.6)
Stroke or TIA, *n* (%)	103 (12.7)	95 (13.8)	6 (7.3)	2 (9.1)	0 (0)
Peripheral vascular disease, *n* (%)	35 (4.3)	32 (4.7)	3 (3.7)	0 (0)	0 (0)
Statin treatment, *n* (%)	265 (32.8)	231 (33.6)	22 (26.8)	6 (27.3)	6 (33.3)
Chronic lung disease, *n* (%) ^1^	89 (11.0)	75 (10.9)	13 (15.8)	0 (0)	1 (5.6)
Active cancer, *n* (%) ^2^	33 (4.1)	29 (4.2)	3 (3.7)	0 (0)	1 (5.6)
Chronic kidney disease, *n* (%) ^3^	164 (20.3)	146 (21.3)	18 (22.0)	0 (0)	0 (0)
Psychotic disorder, *n* (%)	56 (6.9)	42 (6.1)	5 (6.1)	5 (22.7)	4 (22.2)
Mood and anxiety disorders, *n* (%)	92 (11.4)	79 (11.5)	7 (8.5)	5 (22.7)	1 (5.6)
Standard alcohol, *n* (%)	85 (10.5)	75 (10.9)	5 (4.9)	3 (13.6)	3 (16.7)
Alcohol excess, *n* (%) ^4^	91 (11.3)	65 (9.5)	13 (15.9)	6 (27.3)	7 (38.9)
Illicit drug use, *n* (%)	97 (12.0)	75 (10.9)	13 (15.9)	6 (27.3)	3 (16.7)

^1^ Chronic obstructive pulmonary disease, interstitial lung disease, bronchiectasis. ^2^ Excludes cancer in remission not receiving hormonal, chemo-, or immunotherapy. ^3^ Estimated glomerular filtration rate < 60 mL/min/1.73m^2^. ^4^ Drinking > 2 standard drinks per day on average, or drinking > 4 standard drinks per occasion. Abbreviations: TIA, transient ischemic attack; IQR, interquartile range.

**Table 2:** Biochemistry at hospital presentation and relevant clinical data grouped by serum sodium concentration.

		Corrected Serum Sodium (mmol/L)			
Characteristic	Total	135–145	130–134	125–129	<125
	*n* = 809	*n* = 687	*n* = 82	*n* = 22	*n* = 18
Peak CK, median (IQR), × 10^3^ U/L	4.1 (1.9–12.0)	3.9 (1.9–11.2)	4.7 (2.2–12.8)	7.3 (3.6–40.8)	9.4 (2.4–21.2)
Sodium, mean (SD), mmol/L ^1^	138.3 (4.7)	139.8 (2.6)	132.8 (1.4)	127.0 (1.3)	119.6 (3.8)
Osmolality, mean (SD), mOsm/kg ^2^	NA	NA	NA	272 (16)	256 (13)
Potassium, mean (SD), mmol/L	4.29 (0.75)	4.28 (0.75)	4.47 (0.91)	4.18 (0.51)	4.01 (0.68)
Calcium, mean (SD), mmol/L ^3^	2.31 (0.18)	2.33 (0.18)	2.26 (0.17)	2.17 (0.27)	2.16 (0.18)
Phosphate, mean (SD), mmol/L ^3^	1.29 (0.58)	1.27 (0.55)	1.41 (0.77)	1.37 (0.63)	1.36 (0.61)
Magnesium, mean (SD), mmol/L ^3^	0.87 (0.19)	0.88 (0.19)	0.85 (0.19)	0.89 (0.24)	0.89 (0.21)
Glucose, median (IQR), mmol/L	6.6 (5.5–8.3)	6.7 (5.5–8.3)	6.3 (5.4–7.9)	6.6 (5.7–7.8)	6.6 (4.9–8.0)
Creatinine, median (IQR), µmol/L	108 (79–184)	106 (79–180)	125 (92–278)	118 (66–372)	82 (63–112)
Acute kidney injury, *n* (%)	402 (49.7)	336 (48.9)	46 (56.1)	13 (59.1)	7 (39.0)
Renal replacement therapy, *n* (%)	38 (4.7)	31 (4.5)	5 (6.1)	1 (4.6)	1 (5.6)
Sepsis syndrome, *n* (%)	115 (14.2)	92 (13.4)	14 (17.1)	5 (22.7)	4 (22.2)
Hospital LOS, median (IQR), days	7 (4–12)	7 (4–12)	6 (4–13)	9 (5–11)	9 (6–19)
Intensive care admission, *n* (%)	182 (22.5)	141 (20.5)	24 (29.3)	6 (27.3)	11 (61.1)

^1^ Corrected for glucose levels. ^2^ Reference range, 280 to 300 mOsm/kg; ^3^ missing observations, *n* = 57 (7%). Abbreviations: LOS, length of stay; CK, creatine kinase; IQR, interquartile range; SD, standard deviation; NA, data not available.

**Table 3:** Mechanisms of hyponatremia in order of frequency of association (*n* = 40).

	Corrected Serum Sodium (mmol/L)		
Etiology or Contributing Factor	<130	125–129	<125
	*n* = 40	*n* = 22	*n* = 18
Hypovolemia, *n* (%)	20 (50)	11 (50)	9 (50)
Alcohol misuse or beer potomania, *n* (%)	12 (30)	7 (32)	5 (28)
Antipsychotic medication, *n* (%)	12 (30)	7 (32)	5 (28)
Antidepressants, *n* (%)	9 (23)	6 (27)	3 (17)
Syndrome of inappropriate ADH, *n* (%) ^1^	8 (20)	7 (32)	1 (6)
Primary polydipsia, *n* (%)	7 (18)	1 (5)	6 (33)
Illicit drugs, *n* (%)	6 (15)	3 (14)	3 (17)
Diuretics, *n* (%)	5 (13)	3 (14)	2 (11)
Gastrointestinal losses, *n* (%)	3 (8)	1 (5)	2 (11)
Antiepileptics, *n* (%)	3 (8)	3 (14)	0 (0)
Pregabalin, *n* (%)	2 (5)	1 (5)	1 (6)
Cerebral salt wasting, *n* (%)	2 (5)	0 (0)	2 (11)
Heart failure or liver cirrhosis, *n* (%)	2 (5)	0 (0)	2 (11)

Notes: Categories are not mutually exclusive as multiple contributing factors were common. Abbreviations: ADH, antidiuretic hormone; SSRI, selective serotonin reuptake inhibitor; SNRI, selective serotonin–norepinephrine reuptake inhibitor. ^1^ Excludes medication- or drug-induced.

**Table 4:** Mechanisms of rhabdomyolysis grouped by serum sodium concentration.

		Corrected Serum Sodium (mmol/L)			
Characteristic	Total	135–145	130–134	125–129	<125
	*n* = 809	*n* = 687	*n* = 82	*n* = 22	*n* = 18
Pressure, *n* (%)	590 (72.9)	507 (73.8)	59 (72.0)	16 (72.7)	8 (44.4)
Drugs and toxins, *n* (%)	171 (21.1)	141 (20.5)	20 (24.4)	7 (31.8)	5 (27.8)
Infection, *n* (%)	89 (11.0)	72 (10.5)	12 (14.6)	3 (13.6)	2 (11.0)
Trauma, *n* (%)	70 (8.7)	54 (7.9)	11 (13.4)	5 (22.7)	0 (0)
Exertional, *n* (%)	52 (6.4)	48 (7.0)	4 (4.9)	0 (0)	0 (0)
Seizure, *n* (%)	30 (3.7)	21 (3.1)	2 (2.4)	1 (4.6)	6 (33.3)
Ischemia, *n* (%)	27 (3.3)	23 (3.4)	3 (3.7)	4 (4.6)	0 (0)
Thermal extremes, *n* (%)	21 (2.6)	17 (2.5)	3 (3.7)	0 (0)	1 (5.6)
Inflammatory, *n* (%)	7 (0.9)	4 (0.6)	2 (2.4)	0 (0)	1 (5.6)
Inherited, *n* (%)	7 (0.9)	7 (1.0)	0 (0)	0 (0)	0 (0)
Electrolytes, *n* (%) ^1^	6 (0.7)	4 (0)	0 (0)	1 (4.6)	1 (5.6)

Notes: Categories were not mutually exclusive. ^1^ Excluding hyponatremia.

**Table 5:** Univariable regression analysis of log creatine kinase on independent variables.

Variable	*b* coef.	95% CI	*p*-Value
Ordinal, per category change ^1^	0.28	0.07, 0.48	0.009
Binary, Na^+^ <130 mmol/L	0.71	0.14, 1.28	0.015
Age, per 10 years	−0.36	−0.41, −0.31	<0.001
Diabetes mellitus	−0.70	−0.99, −0.40	<0.001
Chronic kidney disease	−0.66	−0.96, −0.35	<0.001
Psychotic disorder	0.76	0.27, 1.25	0.002
Alcohol intake	0.54	0.24, 0.83	0.001
Illicit drug use	0.77	0.39, 1.15	<0.001
Seizures	1.23	0.59, 1.89	<0.001
Pressure injury	−1.23	−1.51, −0.99	<0.001

^1^ Change from one Na^+^ category to the next (more severe) hyponatremia category.

**Table 6:** Multivariable regression analysis of log creatine kinase on independent variables.

Model and Covariates	*b* coef.	95% CI
Main exposure variable: Na^+^, per category change	0.276	0.070, 0.483
Model 1: + age	0.214	0.030, 0.401
Model 2: + age, alcohol excess, illicit drugs	0.245	0.060, 0.435
Model 3: Model 2 + diabetes, psychotic disorder	0.219	0.030, 0.410

## Data Availability

Deidentified data may be available upon reasonable request from the corresponding author, subject to approval by the Monash Health Research Directorate.

## Supplementary Materials

The following supporting information can be downloaded at: https://www.mdpi.com/article/10.3390/jcm11113215/s1, Figure S1: Outcome of logarithmic transformation of peak serum creatine kinase; Figure S2: Regression diagnostic plots for the final multivariable model. Table S1: Description of rhabdomyolysis categories and examples used in the context of the observational study by Lim et al.

## References

[1] Cronin R.E. (1987). Psychogenic polydipsia with hyponatremia: Report of eleven cases. Am. J. Kidney Dis..

[2] Putterman C., Levy L., Rubinger D. (1993). Transient exercise-induced water intoxication and rhabdomyolysis. Am. J. Kidney Dis..

[3] Trimarchi H., Gonzalez J., Olivero J. (1999). Hyponatremia-associated rhabdomyolysis. Nephron.

[4] Kaur J., Kumar D., Alfishawy M., Lopez R., Sachmechi I. (2016). Paliperidone Inducing Concomitantly Syndrome of Inappropriate Antidiuretic Hormone, Neuroleptic Malignant Syndrome, and Rhabdomyolysis. Case Rep. Crit. Care.

[5] Halpern P., Moskovich J., Avrahami B., Bentur Y., Soffer D., Peleg K. (2011). Morbidity associated with MDMA (ecstasy) abuse: A survey of emergency department admissions. Hum. Exp. Toxicol..

[6] Ben-Abraham R., Szold O., Rudick V., Weinbroum A.A. (2003). ‘Ecstasy’ intoxication: Life-threatening manifestations and resuscitative measures in the intensive care setting. Eur. J. Emerg. Med..

[7] Kashiura M., Sugiyama K., Hamabe Y. (2017). Association between rapid serum sodium correction and rhabdomyolysis in water intoxication: A retrospective cohort study. J. Intensive Care.

[8] Cairns R.S., Hew-Butler T. (2016). Proof of concept: Hypovolemic hyponatremia may precede and augment creatine kinase elevations during an ultramarathon. Eur. J. Appl. Physiol..

[9] Peled M., Dolkart O., Finn T., Amar E., Zeltser D. (2014). No association between hyponatremia and rhabdomyolysis in rats. J. Emerg. Med..

[10] Hoffman M.D., Ingwerson J.L., Rogers I.R., Hew-Butler T., Stuempfle K.J. (2012). Increasing creatine kinase concentrations at the 161-km Western States Endurance Run. Wilderness Environ. Med..

[11] Spasovski G., Vanholder R., Allolio B., Annane D., Ball S., Bichet D., Decaux G., Fenske W., Hoorn E.J., Ichai C. (2014). Hyponatraemia Guideline Development Group. Clinical practice guideline on diagnosis and treatment of hyponatraemia. Eur. J. Endocrinol..

[12] Siew E.D., Ikizler T.A., Matheny M.E., Shi Y., Schildcrout J.S., Danciu I., Dwyer J.P., Srichai M., Hung A.M., Smith J.P. (2012). Estimating baseline kidney function in hospitalized patients with impaired kidney function. Clin. J. Am. Soc. Nephrol..

[13] Kellum J.A., Lameire N., Aspelin P., Barsoum R.S., Burdmann E.A., Goldstein S.L., Herzog C.A., Joannidis M., Kribben A., Levey A.S. (2012). Kidney disease: Improving global outcomes (KDIGO) acute kidney injury work group. KDIGO clinical practice guideline for acute kidney injury. Kidney Int. Suppl..

[14] Singer M., Deutschman C.S., Seymour C.W., Shankar-Hari M., Annane D., Bauer M., Bellomo R., Bernard G.R., Chiche J.D., Coopersmith C.M. (2016). The Third International Consensus Definitions for Sepsis and Septic Shock (Sepsis-3). JAMA.

[15] Australian Bureau of Statistics National Health Survey: First Results, 2017–2018. ABS: Canberra, Australia, 2018, ABS cat. no. 4364.0.55.001. https://www.abs.gov.au/statistics/health/health-conditions-and-risks/national-health-survey-first-results/latest-release.

[16] Morgan V.A., McGrath J.J., Jablensky A., Badcock J.C., Waterreus A., Bush R., Carr V., Castle D., Cohen M., Galletly C. (2014). Psychosis prevalence and physical, metabolic and cognitive co-morbidity: Data from the second Australian national survey of psychosis. Psychol. Med..

[17] Australian Institute of Health and Welfare (2021). Illicit Drug Use. AIHW: Canberra, Australia. https://www.aihw.gov.au/reports/australias-health/illicit-drug-use.

[18] Rangan G.K., Dorani N., Zhang M.M., Abu-Zarour L., Lau H.C., Munt A., Chandra A.N., Saravanabavan S., Rangan A., Zhang J.Q. (2021). Clinical characteristics and outcomes of hyponatraemia associated with oral water intake in adults: A systematic review. BMJ Open.

[19] Overgaard-Steensen C., Stødkilde-Jørgensen H., Larsson A., Broch-Lips M., Tønnesen E., Frøkiaer J., Ring T. (2010). Regional differences in osmotic behavior in brain during acute hyponatremia: An in vivo MRI-study of brain and skeletal muscle in pigs. Am. J. Physiol. Regul. Integr. Comp. Physiol..

[20] Cairns S.P., Buller S.J., Loiselle D.S., Renaud J.M. (2003). Changes of action potentials and force at lowered [Na^+^]o in mouse skeletal muscle: Implications for fatigue. Am. J. Physiol. Cell Physiol..

[21] Vandergheynst F., Gombeir Y., Bellante F., Perrotta G., Remiche G., Mélot C., Mavroudakis N., Decaux G. (2016). Impact of hyponatremia on nerve conduction and muscle strength. Eur. J. Clin. Invest..

[22] Chen L.C., Bai Y.M., Chang M.H. (2014). Polydipsia, hyponatremia and rhabdomyolysis in schizophrenia: A case report. World J. Psychiatry.

[23] Palmer B.F., Clegg D.J. (2017). Electrolyte Disturbances in Patients with Chronic Alcohol-Use Disorder. N. Engl. J. Med..

[24] Baj J., Flieger W., Teresiński G., Buszewicz G., Sitarz R., Forma A., Karakuła K., Maciejewski R. (2020). Magnesium, Calcium, Potassium, Sodium, Phosphorus, Selenium, Zinc, and Chromium Levels in Alcohol Use Disorder: A Review. J. Clin. Med..

[25] Hall A.P., Henry J.A. (2006). Acute toxic effects of ‘Ecstasy’ (MDMA) and related compounds: Overview of pathophysiology and clinical management. Br. J. Anaesth..

[26] Faria A.C., Carmo H., Carvalho F., Silva J.P., Bastos M.L., Dias da Silva D. (2020). Drinking to death: Hyponatraemia induced by synthetic phenethylamines. Drug Alcohol Depend..

[27] Morita S., Inokuchi S., Yamamoto R., Inoue S., Tamura K., Ohama S., Nakagawa Y., Yamamoto I. (2010). Risk factors for rhabdomyolysis in self-induced water intoxication (SIWI) patients. J. Emerg. Med..

[28] Akmal M., Bishop J.E., Telfer N., Norman A.W., Massry S.G. (1986). Hypocalcemia and hypercalcemia in patients with rhabdomyolysis with and without acute renal failure. J. Clin. Endocrinol. Metab..

