# Supplementary material for: Observational Study of the Association between Hyponatremia and Rhabdomyolysis in Patients Presenting to Hospital
Source: J Clin Med. 2022 Jun 5;11(11):3215. doi: 10.3390/jcm11113215 (PMC9181719; doi:10.3390/jcm11113215)
Supplement: Supplementary file 1 [file jcm-11-03215-s001.zip › jcm-1719007-supplementary/Figure S1.pdf]

Journal of
Clinical Medicine

MDPI

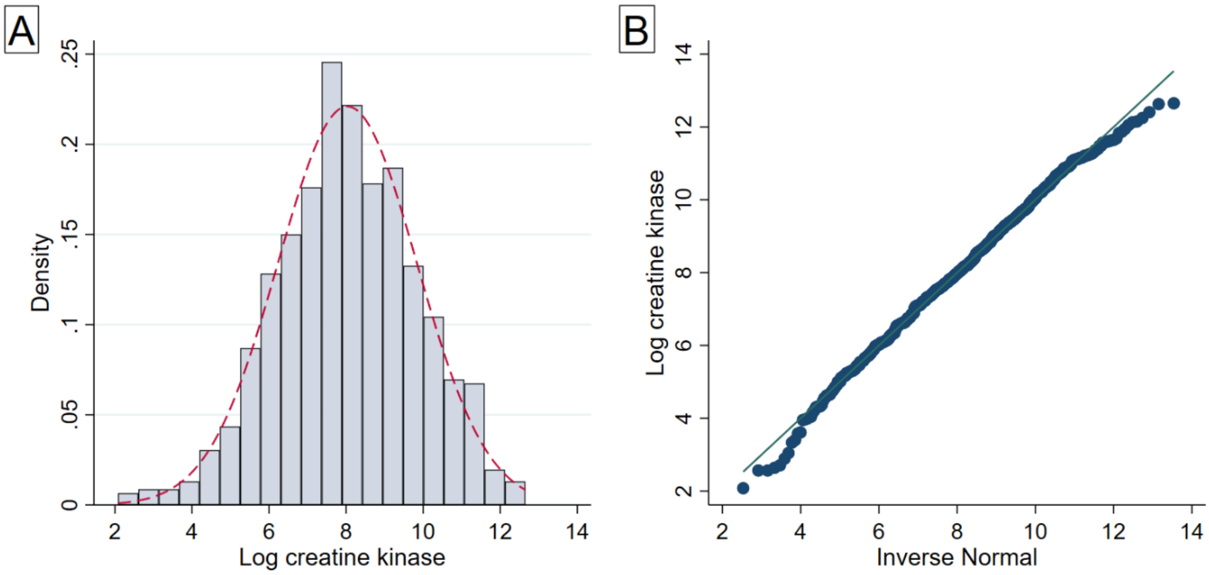

Figure S1. Outcome of logarithmic transformation of peak serum creatine kinase. (A) Histogram of the log transformed creatine kinase values with superimposed normal distribution curve. (B) Normality probability plot (Q-Q plot) showed a good outcome of the transformation.
