# Supplementary material for: Observational Study of the Association between Hyponatremia and Rhabdomyolysis in Patients Presenting to Hospital
Source: J Clin Med. 2022 Jun 5;11(11):3215. doi: 10.3390/jcm11113215 (PMC9181719; doi:10.3390/jcm11113215)
Supplement: Supplementary file 1 [file jcm-11-03215-s001.zip › jcm-1719007-supplementary/Figure S2.pdf]

Journal of
Clinical Medicine

MDPI

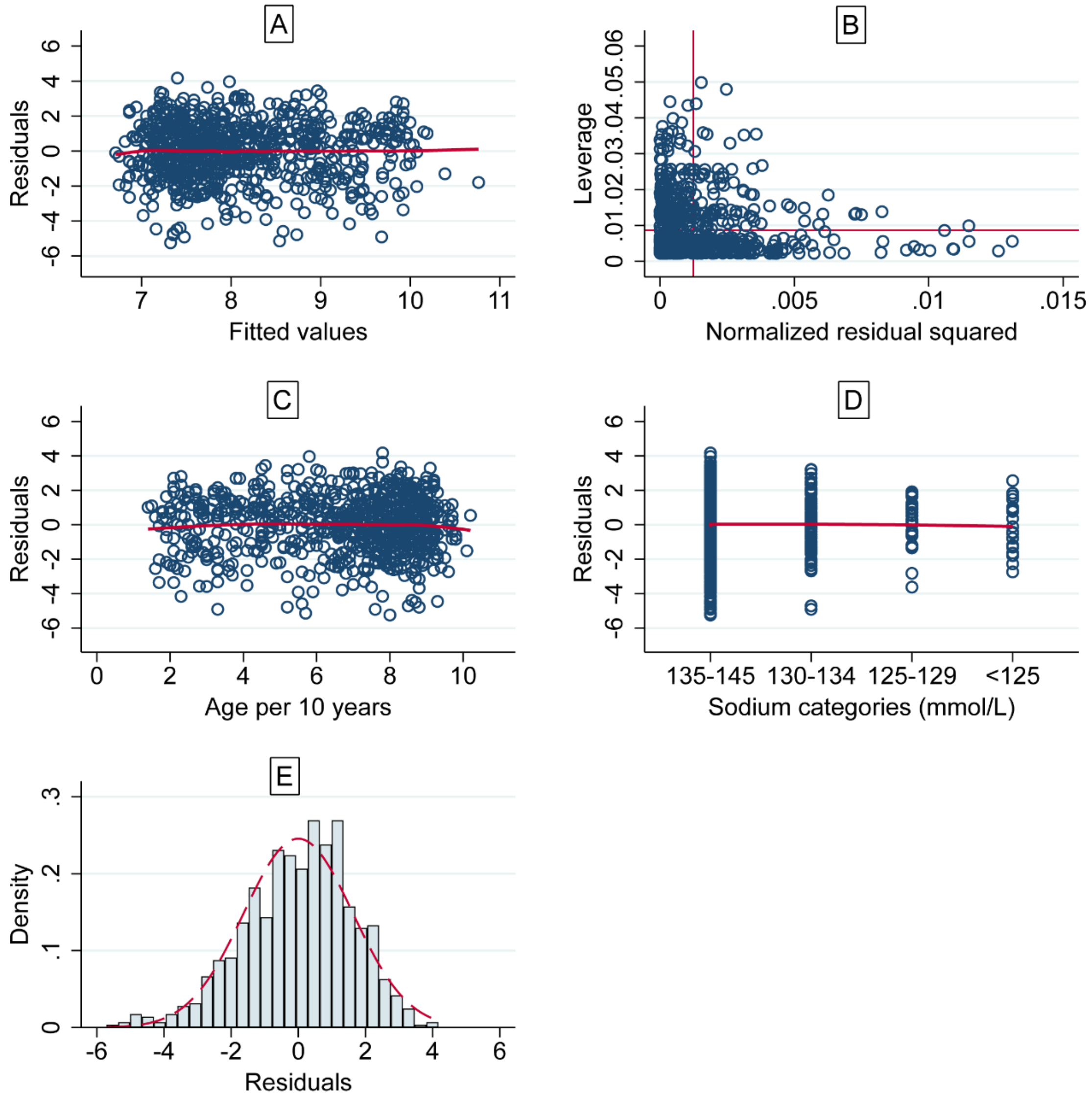

Figure S2. Regression diagnostic plots for the final multivariable model. (A) The residuals versus fitted plot with lowess line (red) showed an even distribution of residuals across the range of fitted values. (B) The leverage versus normalized residual squared plot identified several observations of minor concern which were verified in the data to be valid. (C) The residuals versus the predictor (age) with lowess line (red) showed an even distribution of residuals across the range of fitted values. (D) The residuals versus exposure (sodium categories) with lowess line (red) showed a mostly even distribution of residuals across the categories. (D) Histogram of residuals with normal distribution curve (dashed red) showing the overall residuals of the final multivariable model were normally distributed.
