# Supplementary material for: Observational Study of the Association between Hyponatremia and Rhabdomyolysis in Patients Presenting to Hospital
Source: J Clin Med. 2022 Jun 5;11(11):3215. doi: 10.3390/jcm11113215 (PMC9181719; doi:10.3390/jcm11113215)
Supplement: Supplementary file 1 [file jcm-11-03215-s001.zip › jcm-1719007-supplementary/Table S1.pdf]

Journal of
Clinical Medicine

Table S1. Description of rhabdomyolysis categories and examples used in the context of the observational study by Lim et al.

Category	Description	Examples
Trauma	Direct muscle injury to specific muscle group(s).	Crush injury, motor vehicle accident, fall from above ground height with significant injuries, physical assault or restrain.
Ischemia	Loss or severely reduced blood supply.	Limb arterial occlusion or embolism due to peripheral vascular disease or atrial fibrillation.
Pressure	Nontraumatic prolonged immobilization, usually in recumbent position.	Prolonged lie on the floor or confined space following syncope, reduced conscious state, debility, or ground-level fall.
Exertional	Extreme physical exertion or exercise, excluding seizures.	Marathon, weightlifting, spin class, skiing, cycling, psychotic motor agitation.
Seizures	Prolonged convulsions with motor manifestations.	Recurrent or prolonged tonic-clonic or clonic seizures, or status epilepticus.
Infection	Viral and bacterial infections.	Influenza A and B, herpes simplex, mycoplasma pneumoniae, legionella, streptococcal or staphylococcal toxic shock syndrome (list is not exhaustive).
Drugs & toxins	Prescribed or illicit drugs known to be linked with rhabdomyolysis, toxin exposure, or envenomation.	Statins, heroin, methamphetamine, neuroleptic malignant syndrome with psychotropics, snake bite, alcohol poisoning.
Inflammatory	Inflammatory myopathies.	Polymyositis, dermatomyositis, inclusion body myositis, autoimmune disease-related myositis, paraneoplastic phenomenon.
Electrolytes	Severe electrolyte disorder other than hyponatremia (main exposure variable in this study).	Potassium < 2.5 mmol/L, phosphate < 0.40 mmol/L, magnesium < 0.40 mmol/L.
Thermal extreme	Body temperature >40.5°C, or <32°C, measured by first responders or in Emergency.	Environmental or excessive heat generation, such heat stroke, malignant hyperthermia. Prolonged cold exposure, near-drowning.
Inherited/familial	Known diagnosis of genetic or inherited disease predisposing to muscle breakdown.	Metabolic myopathies, inborn errors of metabolism, mitochondrial disease.
